# Supplementary material for: AlphaFold models of host-pathogen interactions elucidate the prevalence and structural modes of molecular mimicry
Source: bioRxiv. 2025 Jun 6:2025.06.04.657796. Preprint. [Version 1] doi: 10.1101/2025.06.04.657796 (PMC12258915; doi:10.1101/2025.06.04.657796)
Supplement: 1 [file NIHPP2025.06.04.657796v1-supplement-1.pdf]

bioRxiv preprint doi: https://doi.org/10.1101/2025.06.04.657796; this version posted June 6, 2025. The copyright holder for this preprint (which was not certified by peer review) is the author/funder, who has granted bioRxiv a license to display the preprint in perpetuity. It is made available under aCC-BY 4.0 International license.

Supplementary Materials

Supplementary Tables

Table S1 - Benchmark dataset of viral-mammalian and bacterial-mammalian protein pairs selected from PDB used to evaluate the performance of AF-multimer and AF3.

Table S2 - Model quality scores for the AF-multimer structures predicted for the benchmark pairs.

Table S3 - Model quality scores for the AF3 structures predicted for the benchmark pairs.

Table S4 - Dataset of human-pathogen protein pairs selected from AP-MS studies and HPIDB.

bioRxiv preprint doi: https://doi.org/10.1101/2025.06.04.657796; this version posted June 6, 2025. The copyright holder for this preprint (which was not certified by peer review) is the author/funder, who has granted bioRxiv a license to display the preprint in perpetuity. It is made available under aCC-BY 4.0 International license.

Table S5 - Predicted model confidence scores for the AF-multimer structures predicted for the AP-MS and HPIDB pairs.

Table S6 - Predicted model confidence scores for the AF3 structures predicted for the AP-MS and HPIDB pairs.

Table S7 - Human-human protein-protein interaction pairs from (Jänes et al., 2024) used in this study (structures with a pDockQ score of 0.49 or higher).

Table S8 - Pathogen-human and human-human protein-protein interaction pairs considered for interface comparison.

Table S9 - Number of pathogen proteins per human ("bait") protein.

Table S10 - Interface similarity scores calculated between human-pathogen interfaces sharing the same human ("bait") protein.

Table S11 - Interface similarity scores calculated between human-pathogen and human-human interfaces sharing the same human ("bait") protein.

Table S12 - Pathogen interfaces classified by the type of similarity they share with human interfaces. Interfaces were classified considering the human-human pairs that scored the highest in terms of Jaccard index and iAlign iTM-score.

Table S13 - Mimicry of short linear motifs detected among pathogen proteins with linear/less structured interface regions.

Table S14 - Pathogen proteins targeting interfaces not targeted by human proteins.

bioRxiv preprint doi: https://doi.org/10.1101/2025.06.04.657796; this version posted June 6, 2025. The copyright holder for this preprint (which was not certified by peer review) is the author/funder, who has granted bioRxiv a license to display the preprint in perpetuity. It is made available under aCC-BY 4.0 International license.

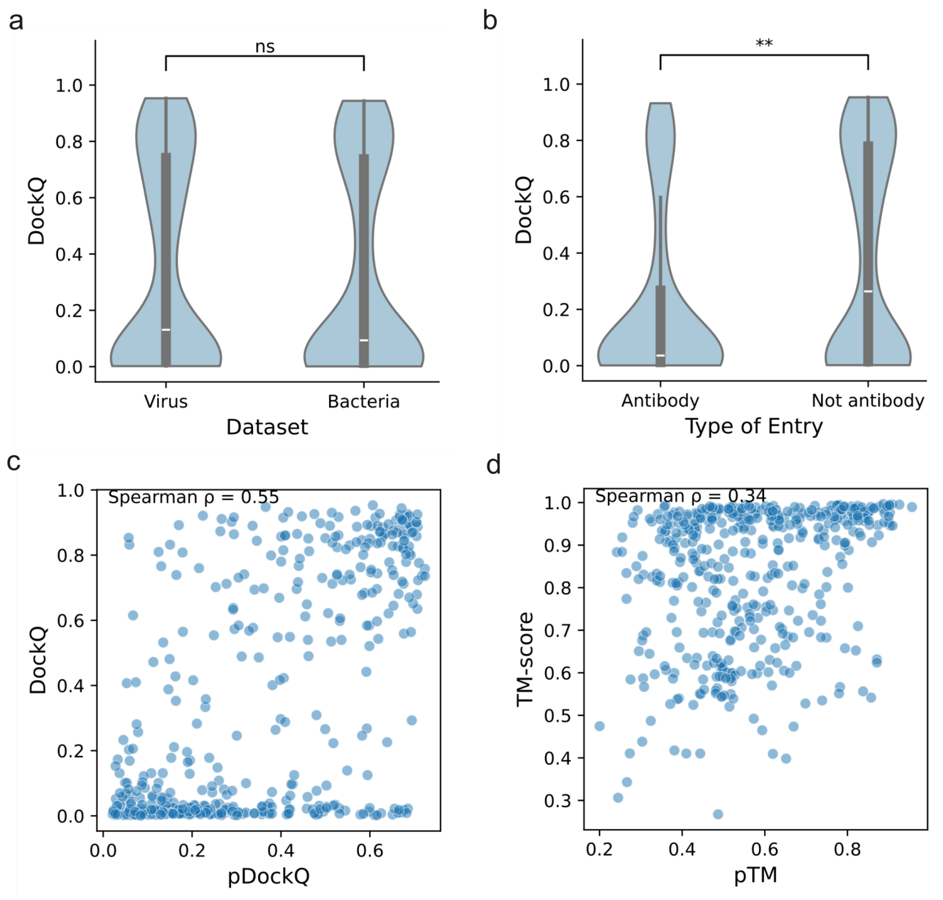

Figure S1 - Results for the benchmark host-pathogen protein pair structures predicted using AF-multimer. a) Comparison between the viral-mammalian and bacteria-mammalian protein pairs in the benchmark dataset, in terms of DockQ scores. DockQ scores are not significantly different between the viral and bacterial protein pairs (Mann-Whitney U test p-value = 0.57). b) Comparison between protein pairs in which one of the interactors is an antibody (N = 119) and pairs without antibodies (N=333). DockQ scores are significantly lower for protein pairs involving antibodies (Mann-Whitney U test p-value = 0.001). c) Correlation between pDockQ (as defined in (Burke et al., 2023)) and DockQ scores for AF-multimer structures (Spearman's ρ = 0.55). d) Correlation between pTM and TM-score values for AF-multimer structures (Spearman's ρ = 0.34).

bioRxiv preprint doi: https://doi.org/10.1101/2025.06.04.657796; this version posted June 6, 2025. The copyright holder for this preprint (which was not certified by peer review) is the author/funder, who has granted bioRxiv a license to display the preprint in perpetuity. It is made available under aCC-BY 4.0 International license.

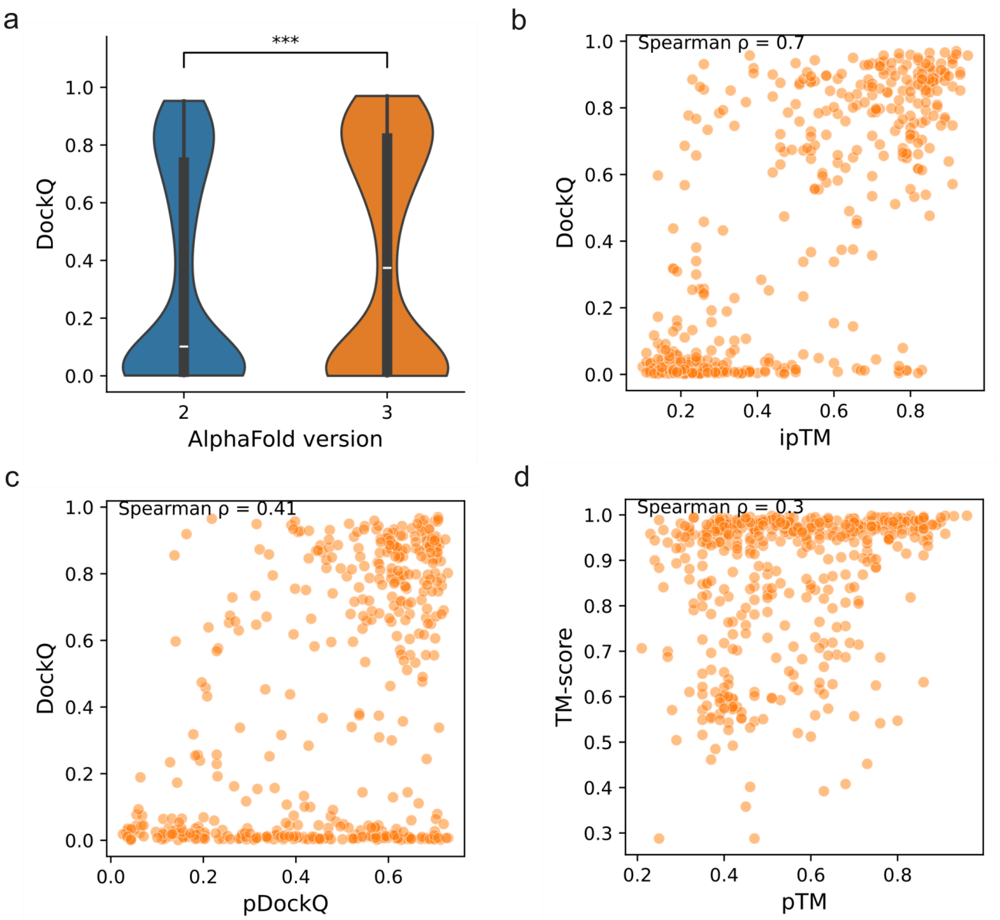

Figure S2 - Results for the benchmark host-pathogen protein pair structures predicted with AF3. a) Comparison of DockQ scores for structures predicted with AF-multimer and AF3. AF3 structures for the benchmark protein pairs have significantly higher DockQ scores than the AF-multimer structures (Wilcoxon signed-rank test p-value = 1.36 x 10^{-5}). b) Correlation between ipTM scores and DockQ scores for AF3 structures (Spearman's ρ = 0.70). c) Correlation between pDockQ (as defined in (Burke et al., 2023)) scores and DockQ scores for AF3 structures (Spearman's ρ = 0.41). d) Correlation between pTM and TM-score values for AF3 structures (Spearman's ρ = 0.30).

bioRxiv preprint doi: https://doi.org/10.1101/2025.06.04.657796; this version posted June 6, 2025. The copyright holder for this preprint (which was not certified by peer review) is the author/funder, who has granted bioRxiv a license to display the preprint in perpetuity. It is made available under aCC-BY 4.0 International license.

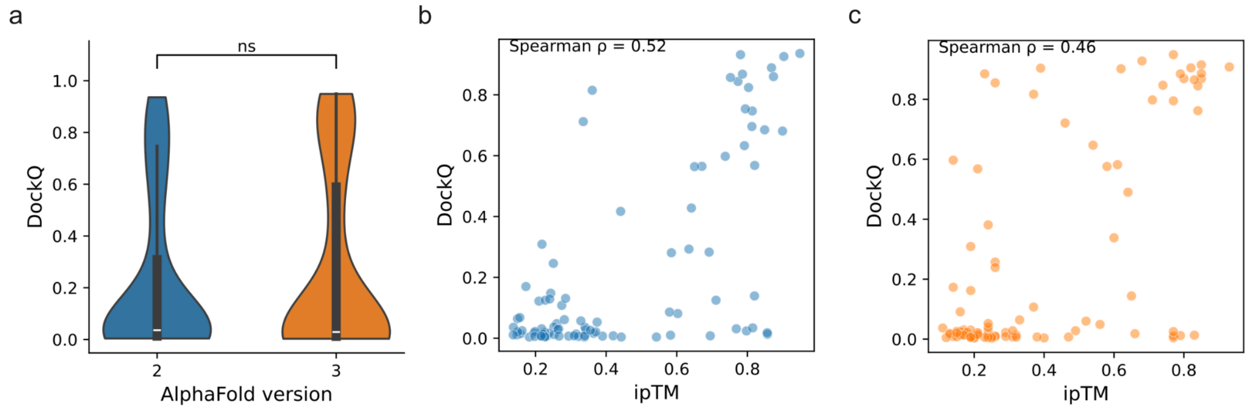

Figure S3 - Results for the benchmark host-pathogen protein pairs that were released after the AF-multimer v. 2.3.0 and AF3 training cutoff date (30 September 2021). a) Comparison of DockQ scores for structures predicted with AF-multimer and AF3. DockQ scores for the protein pairs released after the training cutoff are not significantly different between AF-multimer and AF3 (Wilcoxon signed-rank test p-value = 0.55). b) Correlation between ipTM scores and DockQ scores for structures predicted with AF-multimer, when only considering the benchmark pairs released after the training cutoff date (Spearman's ρ = 0.52). c) Correlation between ipTM scores and DockQ scores for structures predicted with AF3, when only considering the benchmark pairs released after the training cutoff date (Spearman's ρ = 0.46).

bioRxiv preprint doi: https://doi.org/10.1101/2025.06.04.657796; this version posted June 6, 2025. The copyright holder for this preprint (which was not certified by peer review) is the author/funder, who has granted bioRxiv a license to display the preprint in perpetuity. It is made available under aCC-BY 4.0 International license.

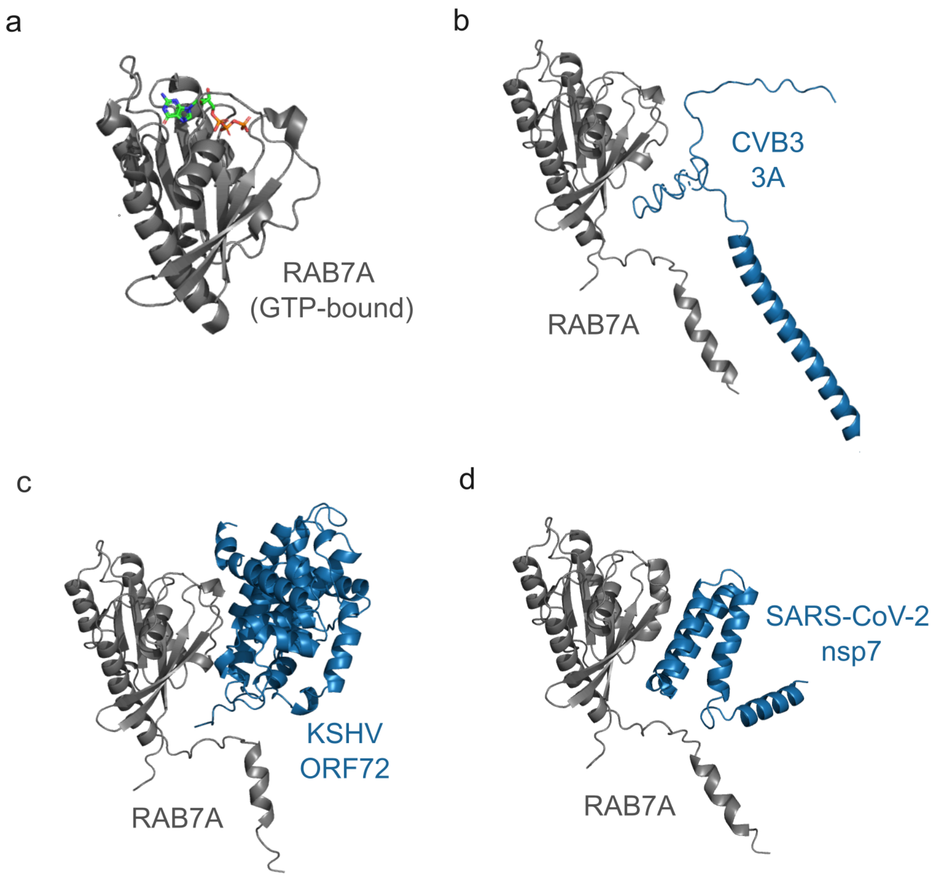

Figure S4 - Comparison between an experimental structure of GTP-bound RAB7A (in gray) and AF-multimer predicted structures for RAB7A interacting with 3 different viral proteins (in blue). a) Crystal structure of RAB7A binding GTP (PDB 1T91 (M. Wu et al., 2005)). b) Predicted structure for the interaction between RAB7A and CVB3 3A. c) Predicted structure for the interaction between RAB7A and KSHV ORF72. d) Predicted structure for the interaction between RAB7A and SARS-CoV-2 nsp7.

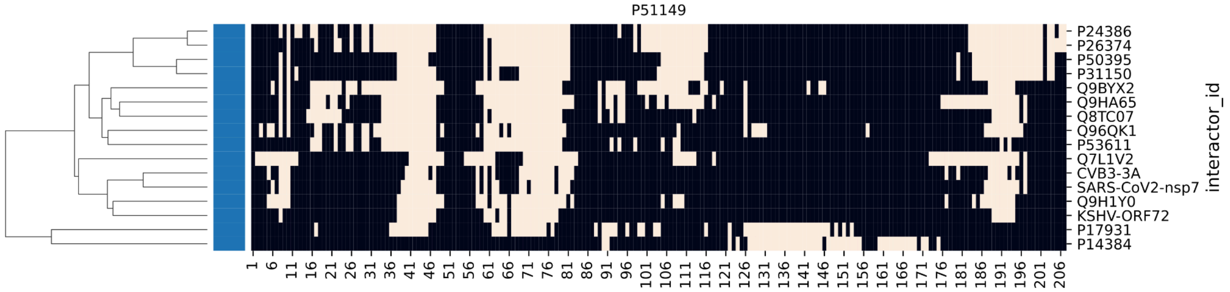

Figure S5 - Residues from Ras-related protein Rab-7a (RAB7A, P51149) targeted by various human and pathogen proteins (interactor_id). Targeted residues are shown in beige, and the remaining residues are shown in black. The targeted interface residues were clustered based on the Jaccard distance.

bioRxiv preprint doi: https://doi.org/10.1101/2025.06.04.657796; this version posted June 6, 2025. The copyright holder for this preprint (which was not certified by peer review) is the author/funder, who has granted bioRxiv a license to display the preprint in perpetuity. It is made available under aCC-BY 4.0 International license.

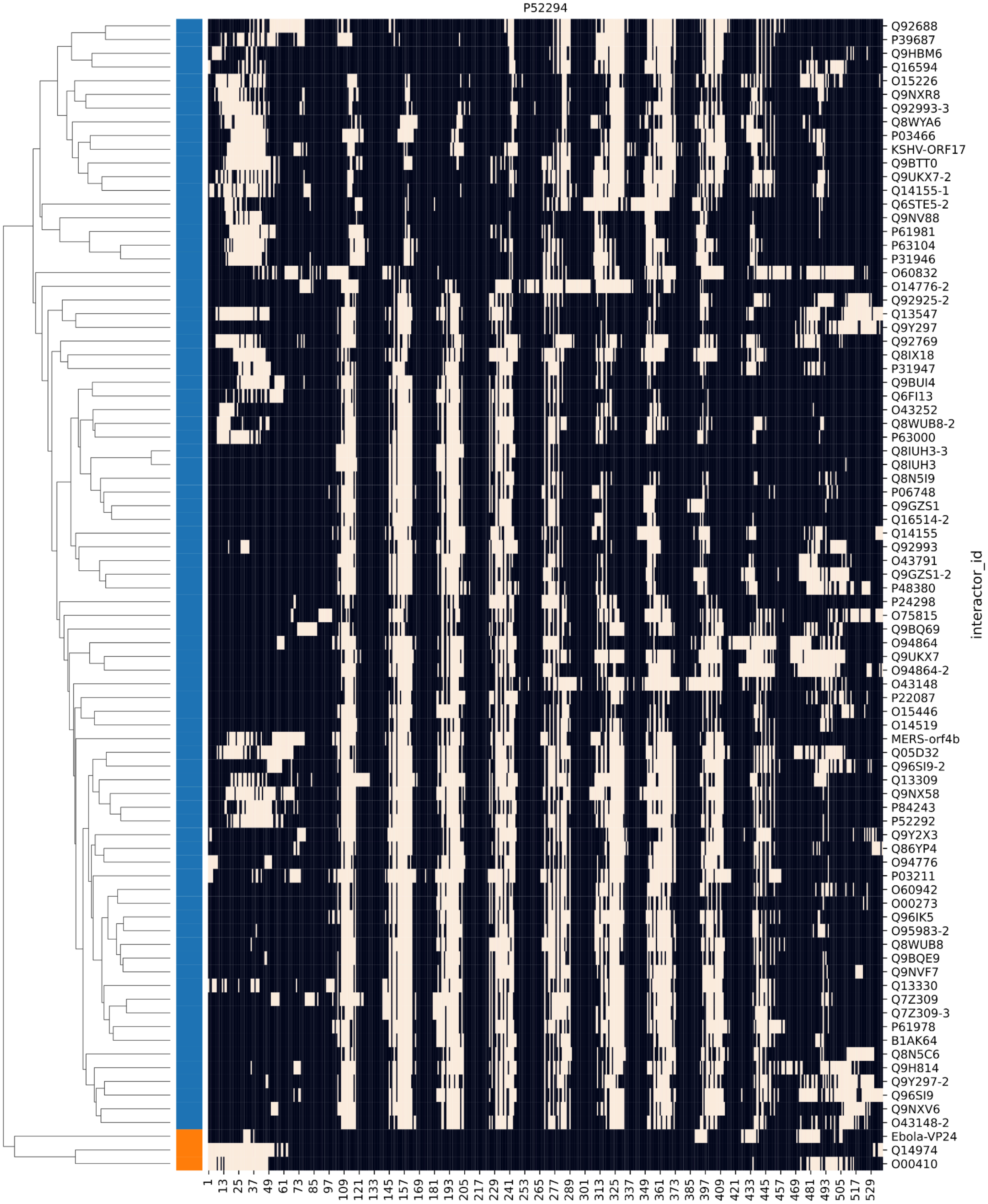

Figure S6 - Residues from Importin subunit alpha-5 (KPNA1, Uniprot ID P52294) targeted by various human and pathogen proteins (interactor_id). Targeted residues are shown in beige,

bioRxiv preprint doi: https://doi.org/10.1101/2025.06.04.657796; this version posted June 6, 2025. The copyright holder for this preprint (which was not certified by peer review) is the author/funder, who has granted bioRxiv a license to display the preprint in perpetuity. It is made available under aCC-BY 4.0 International license.

and the remaining residues are shown in black. The targeted interface residues were clustered based on the Jaccard distance.

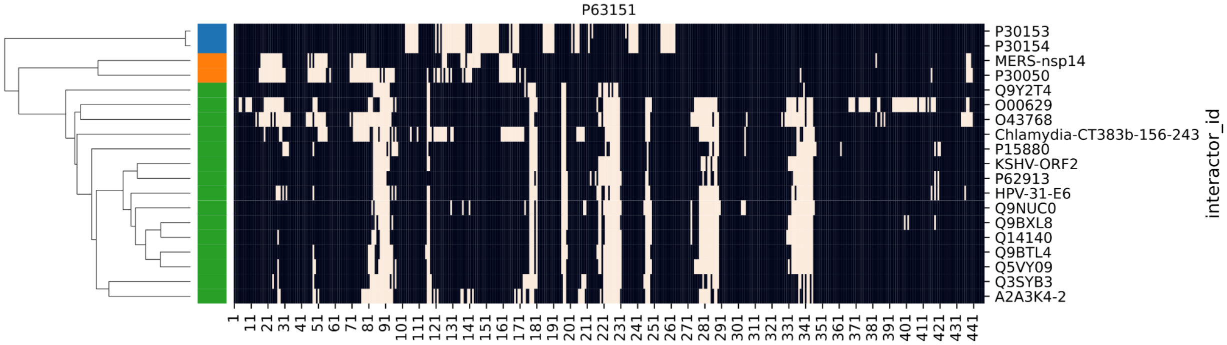

Figure S7 - Residues from Serine/threonine-protein phosphatase 2A 55 kDa regulatory subunit B alpha isoform (PPP2R2A, Uniprot ID P63151) targeted by various human and pathogen proteins (interactor_id). Targeted residues are shown in beige, and the remaining residues are shown in black. The targeted interface residues were clustered based on the Jaccard distance.

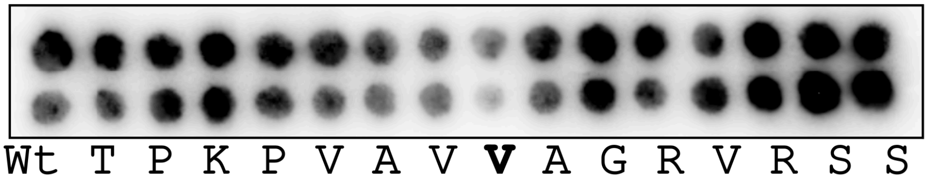

Figure S8 - Alanine scanning experiment for the interaction between SIAH1 and a peptide from the predicted interface residues in KSHV ORF45 (TPKPVAVVAGRVRSS).
